# Socio-Demographic and Geographical Factors in Esophageal and Gastric Cancer Mortality in Sweden

**DOI:** 10.1371/journal.pone.0062067

**Published:** 2013-04-18

**Authors:** Rickard Ljung, Sven Drefahl, Gunnar Andersson, Jesper Lagergren

**Affiliations:** 1 Unit of Upper Gastrointestinal Research, Department of Molecular Medicine and Surgery, Karolinska Institutet, Stockholm, Sweden; 2 Stockholm University SIMSAM Node for Demographic Research, Department of Sociology, Stockholm University, Stockholm, Sweden; 3 Division of Cancer Studies, Kinǵs College London, London, United Kingdom; Okayama University, Japan

## Abstract

**Background:**

Socio-demographic factors and area of residence might influence the development of esophageal and gastric cancer. Large-scale population-based research can determine the role of such factors.

**Methods:**

This population-based cohort study included all Swedish residents aged 30–84 years in 1990–2007. Educational level, marital status, place of birth, and place of residence were evaluated with regard to mortality from esophageal or gastric cancer. Cox regression yielded hazard ratios (HR) with 95% confidence intervals (CI), adjusted for potential confounding.

**Results:**

Among 84 920 565 person-years, 5125 and 12 230 deaths occurred from esophageal cancer and gastric cancer, respectively. Higher educational level decreased the HR of esophageal cancer (HR = 0.61, 95%CI 0.42–0.90 in women, HR = 0.71, 95%CI 0.60–0.84 in men) and gastric cancer (HR = 0.80, 95%CI 0.63–1.03 in women, HR = 0.73, 95%CI 0.64–0.83 in men). Being unmarried increased HR of esophageal cancer (HR = 1.64, 95%CI 1.35–1.99 in women, HR = 1.64, 95%CI 1.50–1.80 in men), but not of gastric cancer. Being born in low density populated areas increased HR of gastric cancer (HR = 1.23, 95%CI 1.10–1.38 in women, HR = 1.37, 95%CI 1.25–1.50 in men), while no strong association was found with esophageal cancer. Living in densely populated areas increased HR of esophageal cancer (HR = 1.31, 95%CI 1.14–1.50 in women, HR = 1.40, 95%CI 1.29–1.51 in men), but not of gastric cancer.

**Conclusion:**

These socio-demographic inequalities in cancer mortality warrant efforts to investigate possible preventable mechanisms and to promote and support healthier lifestyles among deprived groups.

## Introduction

The incidence of esophageal adenocarcinoma has increased over the last decades in high-income countries, including Sweden, whereas the incidence of esophageal squamous cell carcinoma and gastric cancer has decreased.[1], [2] The main risk factors are gastroesophageal reflux and obesity for esophageal adenocarcinoma,[3], [4] tobacco smoking and heavy alcohol drinking for esophageal squamous cell carcinoma ,[5] and infection with Helicobacter pylori (*H. pylori*) for gastric cancer.[6], [7]. Low socioeconomic position has been shown to be associated with an increased risk of all these cancers, regardless of whether it is measured by educational level,[8]–[15] income,[10], [12] occupation,[8], [10]–[13], [15], [16] or material deprivation.[12], [17] Also, those being single might be at increased risk compared to cohabitants.[8] Moreover, the population density at birth and current residence might influence the development of esophageal and gastric cancer. As these cancers have a very low survival the mortality rate is a good proxy for the incidence rate. There is a need for large-scale population-based research to establish the role of these socio-demographic factors and risk of esophageal and gastric cancer. Therefore, with the aim of clarifying the association between educational level, marital status, place of birth, and place of residence and mortality of esophageal and gastric cancer, we conducted a nationwide Swedish cohort study with follow-up of nearly 85 million person-years.

## Materials and Methods

### Ethics statement

The Regional Ethical Review Board in Stockholm, Sweden approved the study.

### Design

A nationwide cohort study was performed on Sweden during January 1, 1990 – December 31, 2007. All persons aged between 30 and 84 years were followed up for risk of esophageal and gastric cancer mortality, as recorded in the National Causes of Death Register. Information on the highest educational level achieved was obtained from the National Education Register. Information on place of birth and on marital status and place of residence for all years since start of follow-up was obtained from the Register of the Total Population. Study subjects were followed for esophageal and gastric cancer mortality. End of study was: i) Dec 31^st^ 2007, ii) age 85, iii) death, or iv) emigration, whichever occurred first. To censor for person-time no longer at risk of esophageal and gastric cancer death, information on dates of emigration and date of any death was obtained from the Register of the Total Population. The unique personal identity number, a 10-digit number assigned to all Swedish residents, was used to link information within the nationwide registers.[18]

### Registers used for the data collection

*The Causes of Death Register* contains information on date of death for all deceased Swedish residents since 1952 and has a 99.2 % completeness of causes of death.[19]

*The National Education Register* was established by Statistics Sweden in 1985 and is annually updated with information on the highest formal education attained by each individual, from elementary to post-graduate levels.[20]

*The Register of the Total Population* contains individual characteristics on all legal residents in Sweden since 1968 onwards, including data on sex, date and place of birth, marital status, and place of residence.[21]

### Exposure data

*Educational level* was classified into six categories: i) primary and lower secondary education less than 9 years, ii) 9-year compulsory school, iii) upper secondary education less than 3 years, iv) upper secondary education 3 years or more, v) post-secondary education less than 3 years, or vi) post-secondary education 3 years or more.

*Marital status* was classified into four categories: i) married (including same sex partnership), ii) single, iii) divorced, or iv) widowed. As cohabitation is not registered, the latter three categories also comprise people living in non-marital cohabitation. Data on marital status was updated monthly.

*Place of birth* was classified into three categories: i) low-densely populated areas (Northern Sweden: *Norrland*), ii) intermediate-densely populated areas (Non-metropolitan Southern Sweden), or iii) high-densely populated areas (metropolitan Sweden: the *Stockholm, Gothenburg*, and *Malmö* areas).

*Place of residence* was classified in the same way as place of birth. In allocating person-time to place of residence, this status was updated on an annual basis.

### Outcome data

Codes from the 9^th^ and 10^th^ version of the International Classification of Diseases (ICD-9, ICD-10) were used to define the underlying cause of death from esophageal and gastric cancer in the Causes of Death Register. Esophageal cancer was defined by the ICD-9 code 150 and the ICD-10 code C15. It was not possible to distinguish between the main histological types of esophageal cancer, adenocarcinoma and squamous-cell carcinoma, since the Causes of Death Register does not contain such information. Gastric cancer was defined by the ICD-9 code 151 and the ICD-10 code C16. Mortality in these cancers was used as a good proxy for the incidence, as the survival is very low in these cancers.

### Statistical analysis

Cox regression yielded hazard ratios (HR) for covariates of esophageal and gastric cancer mortality with 95 % confidence intervals (CI). Data on death occurrences and marital transition were calculated with the accuracy of a month, educational level, place of birth, and place of residence were calculated with the accuracy of a year. Follow-up was divided into four time periods: i) 1990–1994, ii) 1995–1999, iii) 2000–2004, or iv) 2005–2007. Separate models were estimated for women and men. For each covariate a separate category for missing information was used in the analyses. The adjusted model included adjustment for age and calendar period, and all of the studied exposure variables.

## Results

### Study participants

Table 1 provides sex-specific data on person-years and number of deaths from esophageal cancer and gastric cancer by educational level, marital status, place of birth, and place of residence. The total number of person-years of follow-up was 84 920 565. In 43 217 162 person-years at risk in women, there were 1337 deaths from esophageal cancer (3.1 per 100 000 person-years) and 4629 deaths from gastric cancer (10.8 per 100 000 person-years). In 41 703 403 person-years at risk in men, there were 3788 deaths from esophageal cancer (9.1 per 100 000 person-years) and 7601 deaths from gastric cancer (18.2 per 100 000 person-years).

**Table 1:** Descriptive data, person-years, and deaths from esophageal and gastric cancer in persons 30–84 years of age in Sweden, 1990–2007.

		Esophageal cancer		Gastric cancer	
	Person- years	Deaths	Rate per 100,000	Deaths	Rate per 100,000
**WOMEN**					
***Educational level***					
Primary and lower secondary education<9 years	9 271 573	535	5.8	1 963	21.2
≥9-year compulsory school	3 593 970	107	3.0	275	7.7
Upper secondary education<3 years	13 271 438	272	2.0	894	6.7
Upper secondary education ≥3 years	3 757 309	49	1.3	103	2.7
Post-secondary education<3 years	4 661 906	45	1.0	162	3.5
Post-secondary education ≥3 years	6 296 039	60	1.0	161	2.6
Missing information	2 364 927	269	11.4	1 071	45.3
***Marital status***					
Married	22 985 694	471	2.0	1 916	8.3
Single	8 042 361	139	1.7	389	4.8
Divorced	5 805 048	201	3.5	555	9.6
Widowed	5 570 579	477	8.6	1 612	28.9
Missing information	813 480	49	6.0	157	19.3
***Place of birth***					
Non-metropolitan Southern Sweden	25 769 502	758	2.9	2 764	10.7
Northern Sweden	6 306 999	216	3.4	882	14.0
Metropolitan Sweden	10 900 962	362	3.3	978	9.0
Missing information	239 699	1	0.4	5	2.1
***Place of residence***					
Non-metropolitan Southern Sweden	25 830 786	702	2.7	2 596	10.1
Northern Sweden	4 628 032	132	2.9	595	12.9
Metropolitan Sweden	11 952 075	414	3.5	1 084	9.1
Missing information	806 269	89	11.0	354	43.9
**MEN**					
***Educational level***					
Primary and lower secondary education<9 years	9 138 009	1679	18.4	3271	35.8
≥9-year compulsory school	3 969 076	169	4.3	297	7.5
Upper secondary education<3 years	11 586 394	688	5.9	1200	10.4
Upper secondary education≥3 years	5 580 192	399	7.2	629	11.3
Post-secondary education<3 years	4 236 001	134	3.2	276	6.5
Post-secondary education≥3 years	5 499 008	215	0.0	326	0.0
Missing information	1 694 722	504	29.7	1602	94.5
***Marital status***					
Married	23 692 995	2057	8.7	4691	19.8
Single	11 173 207	654	5.9	1012	9.1
Divorced	4 736 518	631	13.3	753	15.9
Widowed	1 469 718	393	26.7	945	64.3
Missing information	630 965	53	8.4	200	31.7
***Place of birth***					
Intermediate-densely populated areas	24 872 532	2235	9.0	4510	18.1
Low-densely populated areas	5 998 916	529	8.8	1495	24.9
High-densely populated areas	10 664 477	1022	9.6	1588	14.9
Missing information	167 477	2	1.2	8	4.8
***Place of residence***					
Intermediate-densely populated areas	25 204 884	2116	8.4	4439	17.6
Low-densely populated areas	4 661 594	362	7.8	1054	22.6
High-densely populated areas	11 215 371	1120	10.0	1526	13.6
Missing information	621 554	190	30.6	582	93.6

### Socio-demographic factors and risk of esophageal cancer mortality

#### Educational level

The adjusted HR indicated a decreased risk of esophageal cancer mortality with increasing educational attainment in both sexes (Table 2). Compared with upper secondary education 3 years or more, the HR for post-secondary education 3 years or more was 0.61 (95%CI 0.42–0.90) in women and 0.71 (95%CI 0.60–0.84) in men.

**Table 2:** Hazard ratio (HR) and 95 % confidence intervals (95% CI) of esophageal cancer mortality in women and men, 30–84 years of age in Sweden, 1990–2007.

	Esophageal cancer mortality			
	Women		Men	
	HR	95% CI	HR	95% CI
***Educational level***				
Primary and lower secondary education<9 years	0.89	0.66–1.21	1.29	1.15–1.45
≥9-year compulsory school	1.07	0.76–1.50	1.06	0.89–1.27
Upper secondary education less than 3 years	0.80	0.59–1.08	1.13	1.00–1.28
Upper secondary education 3 years or more	1	(reference)	1	(reference)
Post-secondary education less than 3 years	0.59	0.40–0.89	0.68	0.56–0.83
Post-secondary education 3 years or more	0.61	0.42–0.90	0.71	0.60–0.84
***Marital status***				
Married	1	(reference)	1	(reference)
Single	1.64	1.35–1.99	1.64	1.50–1.80
Divorced	1.56	1.32–1.85	1.69	1.54–1.85
Widowed	1.20	1.04–1.38	1.22	1.09–1.36
***Place of birth***				
Intermediate-densely populated areas	1	(reference)	1	(reference)
Low-densely populated areas	1.18	0.96–1.46	0.98	0.85–1.13
High-densely populated areas	1.14	1.00–1.31	1.12	1.03–1.21
***Place of residence***				
Intermediate-densely populated areas	1	(reference)	1	(reference)
Low-densely populated areas	0.94	0.73–1.22	0.94	0.80–1.11
High-densely populated areas	1.31	1.14–1.49	1.40	1.29–1.51

The HRs for the studied exposures are adjusted for age, period, and of each other. The HRs for the categories of missing information is not presented.

#### Marital status

An inverse association of being married was indicated compared with being non-married in both women and men (Table 2). Compared to married persons, the risk of esophageal cancer mortality was increased to a similar extent in single women (HR 1.64, 95%CI 1.35–1.99) and in single men (HR 1.64, 95%CI 1.50–1.80).

#### Place of birth

Being born in a highly densely populated area was associated with a slightly increased risk of esophageal cancer mortality (HR 1.14, 95%CI 1.00–1.31 in women; HR = 1.12, 95%CI 1.03–1.21 in men), compared with being born in an intermediately densely populated area.

#### Place of residence

Living in a highly densely populated area was associated with an increased risk of esophageal cancer mortality compared with living in an intermediately densely populated area (HR = 1.31, 95%CI 1.14–1.50 in women; HR = 1.40, 95%CI 1.29–1.51 in men).

Stratification by age groups (30–64 or 65–84 years) yielded similar results for exposures at working and post-working ages as in the main analysis (data not shown). Stratification by time period showed increasing trends with more recent calendar period in esophageal cancer mortality for men and more stable levels for women (data not shown).

### Socio-demographic factors and risk of gastric cancer mortality

#### Education

The adjusted HR indicated a decreased risk of gastric cancer mortality with increasing educational attainment in both sexes. The HR for post-secondary education 3 years or more was 0.80 (95%CI 0.63–1.03) in women and 0.73 (95%CI 0.64–0.83) in men, compared with having upper secondary education 3 years or more.

#### Marital status

The HR indicated a slightly inverse association of being married compared with being non-married (Table 3). Compared with those being married, the HR for being single was 1.04 (95%CI 0.93–1.16) in women and 1.11 (95%CI 1.04–1.19) in men.

**Table 3:** Hazard ratio (HR) and 95 % confidence intervals (95% CI) of gastric cancer mortality in women and men, 30–84 years of age in Sweden, 1990–2007.

	Gastric cancer mortality				
	Women		Men		
	HR	95% CI	HR	95% CI	
***Educational level***					
Primary and lower secondary education<9 years	1.47	1.20–1.80	1.32	1.21–1.44	
≥9-year compulsory school	1.32	1.05–1.65	1.26	1.10–1.45	
Upper secondary education less than 3 years	1.24	1.01–1.52	1.20	1.08–1.32	
Upper secondary education 3 years or more	1	(reference)	1	(reference)	
Post-secondary education less than 3 years	1.02	0.80–1.31	0.90	0.78–1.04	
Post-secondary education 3 years or more	0.80	0.63–1.03	0.73	0.64–0.83	
***Marital status***					
Married	1	(reference)	1	(reference)	
Single	1.04	0.93–1.16	1.11	1.04–1.19	
Divorced	1.14	1.03–1.25	1.03	0.95–1.11	
Widowed	0.91	0.85–0.98	1.03	0.96–1.11	
***Place of birth***					
Intermediate-densely populated areas	1	(reference)	1	(reference)	
Low-densely populated areas	1.23	1.10–1.38	1.37	1.25–1.50	
High-densely populated areas	0.96	0.89–1.04	1.03	0.97–1.09	
***Place of residence***					
Intermediate-densely populated areas	1	(reference)	1	(reference)	
Low-densely populated areas	1.09	0.96–1.25	0.98	0.88–1.09	
High-densely populated areas	1.02	0.94–1.10	0.94	0.88–1.00	

The HRs for the studied exposures are adjusted for age, period, and of each other. The HRs for the categories of missing information is not presented.

#### Place of birth

Being born in a low-densely populated area (Northern Sweden) indicated an increased risk of gastric cancer mortality, compared to being born in an intermediately densely populated area (HR 1.23, 95%CI 1.10-1.38 in women; HR 1.37, 95%CI 1.25–1.50 in men).

#### Place of residence

There was no increased HR of gastric cancer mortality among persons living in a low densely populated area compared with those living in an intermediately densely populated area (HR 1.09, 95%CI 0.96–1.25 in women; HR 0.98, 95%CI 0.88–1.09 in men).

Stratification by age (30–64 or 65–84) yielded similar results for exposures at working and post-working ages as in the main analysis (data not shown). Stratification by time period showed decreasing trends with more recent calendar period in gastric cancer mortality for both women and men (data not shown).

## Discussion

This study shows that persons with shorter time of schooling, and those being non-married have an increased risk of both esophageal and gastric cancer mortality, while persons born in low densely populated areas have an increased risk of gastric cancer mortality, and those living in highly densely populated areas have an increased risk of esophageal cancer mortality.

Strengths of the study include the cohort design, the large sample size, the complete nationwide coverage of the study exposures (educational attainment, marital status, place of birth, and place of residence) and outcomes (esophageal or gastric cancer mortality). There are, however, also weaknesses, three of which might be of particular relevance. First, we did not have access to cancer incidence data, but must rely on cause-specific cancer death data. Using causes of death data to estimate cancer incidence can introduce a bias as the underlying cause of death is subject to other circumstances than just the concomitant cancer. However, for cancers like esophageal and gastric cancer with a low survival (5-year survival 10–20%), cancer mortality can be regarded a good proxy of the incidence, and perhaps even be more valid than cancer incidence data as those dying shortly after first symptoms (which is not uncommon in these tumors) may only be reported to the Causes of Death Register and not to the Cancer Register.[22], [23] Therefore, the findings regarding risk of esophageal and gastric cancer mortality would be representative of risk of esophageal and gastric cancer incidence for the studied exposures. Secondly, there was no information on some potential confounding factors that could influence the incidence of these cancers, like tobacco smoking, obesity and heavy alcohol drinking. These lifestyle risk factors are more common among individuals in low socioeconomic positions. However, the construct of social stratification in society is of public health importance, and these lifestyle factors could be regarded as being mediating factors on the pathway from social adversity to development of cancer. Hence, if regarded as mediating factors they should not be adjusted for. Factors related to survival from these cancers, like tumor stage, treatment and co –morbidities, would confound the analyses to a very little extent as the mortality in these cancers is very high and the cancer is thus recorded as the cause of death. Thirdly, and finally, the Causes of Death Register contains no information on histological type of the tumors and cannot differentiate between adenocarcinoma and squamous cell carcinoma of the esophagus. With regard to known socio-demographic risk factors, they are however very similar between these two types of esophageal cancer.[8]–[11]

The finding of an inverse association between educational attainment and risk of esophageal and gastric cancer deserves further attention. An inverse association between socioeconomic position and these cancers has been seen in other settings, regardless of whether the measurement of socioeconomic position was educational level, income, or occupation.[8]–[17] However, the present study is the by far largest ever addressing this important topic, and the large study size allows for robust subgroup analyses. There was a clear educational gradient with the highest risk for those with the fewest years of schooling and the lowest risk for those with 15 years or more of study. Educational level is widely used as an indicator of socioeconomic position, in part due to that it is easy to measure, stable in adulthood, and not affected by changes in health status, it is also applicable to persons outside the labor force.[24] The association of a clear gradient in educational attainment and the risk of these cancers can at least partly be due to an increased general awareness of health, including differential exposure to lifestyle risk factors for these cancers, e.g. tobacco smoking and obesity.

Being married is associated with better health in general.[25], [26] For the un-married and divorced the relative risk of esophageal cancer mortality was clearly higher than for gastric cancer mortality. This might partly reflect higher tobacco and heavy alcohol drinking among the non-married, exposures with a particularly strong link with esophageal squamous cell carcinoma. Nevertheless, living a single life is a marker of an increased risk of esophageal and gastric cancer.

The increased risk of gastric cancer among those born in low densely populated areas is probably to a great extent explained by a higher prevalence of *H. pylori* infection, which has been shown for those living in rural areas and in lower socioeconomic position during childhood.[27] Also the decreasing trend in gastric cancer mortality by time is consistent with an increasing socioeconomic standard in general in Sweden over these decades. The higher risk of esophageal cancer mortality among persons living in highly densely populated areas could in part be explained by a lower prevalence of *H. pylori* infection, since this infection seems to decrease the risk of esophageal adenocarcinoma.[28] Also, a higher exposure to environmental factors in metropolitan areas, e.g., air pollution, could be one explanation, but this has not been verified.[29]–[31]

In conclusion, this cohort study of the whole Swedish adult population reveals a clear inverse gradient in educational attainment and risk of esophageal and gastric cancer. Furthermore, the non-married have a higher risk of esophageal cancer. Persons being born in low densely populated areas have a marked increased risk of gastric cancer, while those living in highly densely populated areas have an increased risk of esophageal cancer. These socio-demographic inequalities in cancer mortality warrant efforts to investigate possible preventable mechanisms and to promote and support healthier lifestyles among deprived groups.
